# Deep-Emotion: Facial Expression Recognition Using Attentional Convolutional Network

**DOI:** 10.3390/s21093046

**Published:** 2021-04-27

**Authors:** Shervin Minaee, Mehdi Minaei, Amirali Abdolrashidi

**Affiliations:** 1 Snapchat Inc., Santa Monica, CA 90405, USA; 2 CS Department, Sama Technical College, Azad University, Tonekabon 46817, Iran; mehdiminaei1376@gmail.com; 3 CS Department, University of California, Riverside, CA 92521, USA; amirali.abdolrashidi@email.ucr.edu

**Keywords:** convolutional neural network, attention mechanism, spatial transformer network, facial expression recognition

## Abstract

Facial expression recognition has been an active area of research over the past few decades, and it is still challenging due to the high intra-class variation. Traditional approaches for this problem rely on hand-crafted features such as SIFT, HOG, and LBP, followed by a classifier trained on a database of images or videos. Most of these works perform reasonably well on datasets of images captured in a controlled condition but fail to perform as well on more challenging datasets with more image variation and partial faces. In recent years, several works proposed an end-to-end framework for facial expression recognition using deep learning models. Despite the better performance of these works, there are still much room for improvement. In this work, we propose a deep learning approach based on attentional convolutional network that is able to focus on important parts of the face and achieves significant improvement over previous models on multiple datasets, including FER-2013, CK+, FERG, and JAFFE. We also use a visualization technique that is able to find important facial regions to detect different emotions based on the classifier’s output. Through experimental results, we show that different emotions are sensitive to different parts of the face.

## 1. Introduction

Emotions are an inevitable part of interpersonal communication. They can be expressed in many different forms, which may or may not be observed with the naked eye. Therefore, with the right tools, any indications preceding or following them can be subject to detection and recognition. There has been an increase in the need to detect a person’s emotions in the past few years and increasing interest in human emotion recognition in various fields including, but not limited to, human–computer interfaces [1], animation [2], medicine [3,4], security [5,6], diagnostics for Autism Spectrum Disorders (ASD) in children [7], and urban sound perception [8].

Emotion recognition can be performed using different features, such as facial expressions [2,9,10], speech [5,11], EEG [12], and even text [13]. Among these features, facial expressions are one of the most popular, if not the most popular, due to a number of reasons; they are visible, they contain many useful features for emotion recognition, and it is easier to collect a large dataset of faces (than other means for human recognition) [2,14,15].

Recently, with the use of deep learning and especially convolutional neural networks (CNNs) [16], many features can be extracted and learned for a decent facial expression recognition system [17,18]. It is, however, noteworthy that, in the case of facial expressions, many of the clues come from a few areas of the face, e.g., the mouth and eyes, whereas other parts, such as the ears and hair, play little parts in the output [19]. This means that, ideally, the machine learning framework should focus only on important parts of the face and should be less sensitive to other facial regions.

**Figure 1:**
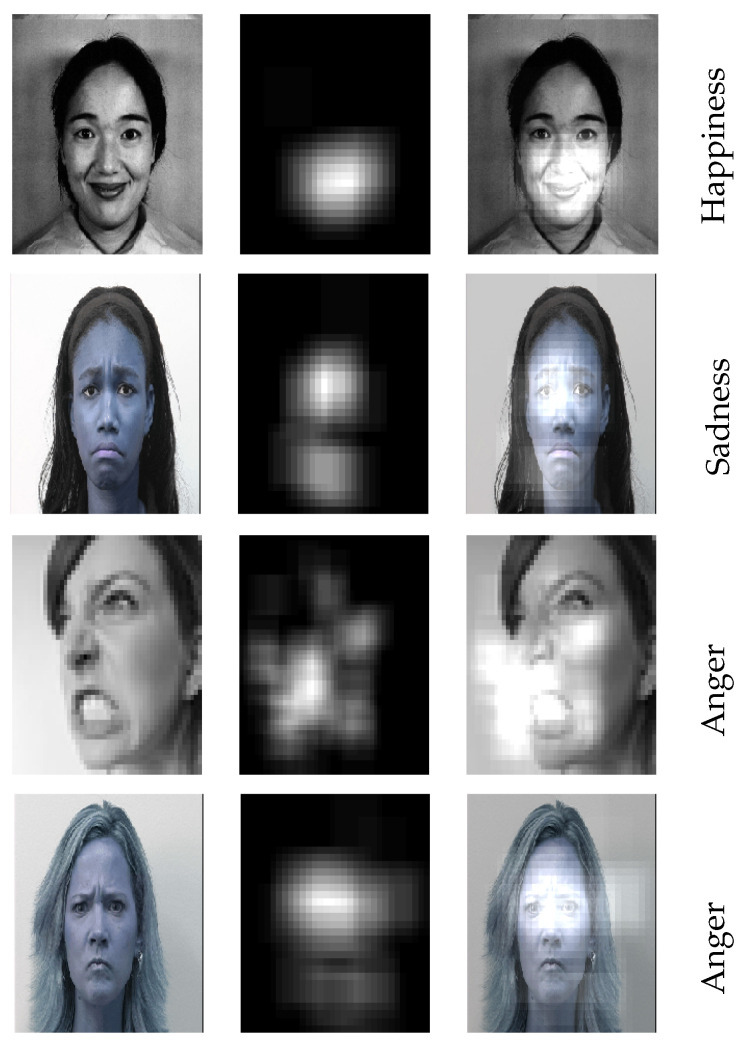
The detected salient regions for different facial expressions using our model. The images in the first and third rows are taken from the FER dataset, and the images in the second and fourth rows belong to the extended Cohn-Kanade dataset.

In this work, we propose a deep learning-based framework for facial expression recognition, which takes the above observation into account and uses an attention mechanism to focus on the salient part of the face. We show that, by using attentional convolutional network, even a network with a few layers (less than 10 layers) is able to achieve a very high accuracy rate. More specifically, this paper presents the following contributions:

- We propose an approach based on an attentional convolutional network, which can focus on feature-rich areas of the face yet remarkably outperforms recent works in accuracy.
- In addition, we use the visualization technique proposed in [20] to highlight the facial image’s most salient regions, i.e., the parts of the image that have the strongest impact on the classifier’s outcome. Samples of salient regions for different emotions are shown in Figure 1.

In the following sections, we first provide an overview of related works in Section 2. The proposed framework and model architecture are explained in Section 3. We then provide the experimental results, overview of databases used in this work, and model visualization in Section 4. Finally, we conclude the paper in Section 5.

## 2. Related Works

**Figure 2:**
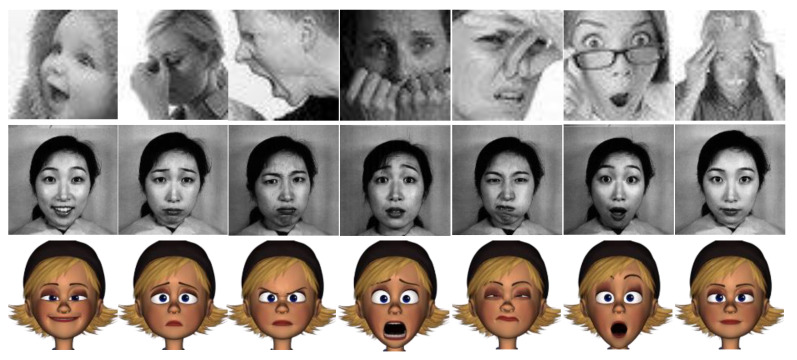
(Left to right) The six cardinal emotions (happiness, sadness, anger, fear, disgust, and surprise) and neutral. The images in the first, second, and the third rows belong to the FER, JAFFE, and FERG datasets, respectively.

In one of the most iconic works in emotion recognition by Paul Ekman [21], happiness, sadness, anger, surprise, fear, and disgust were identified as the six principal emotions (besides neutral). Ekman later developed FACS [22] using this concept, thus setting the standard for work on emotion recognition ever since. Neutral was also included later on in most human recognition datasets, resulting in seven basic emotions. Image samples of these emotions from three datasets are displayed in Figure 2.

Earlier works on emotion recognition relied on the traditional two-step machine learning approach, where in the first step, some features are extracted from the images and, in the second step, a classifier (such as SVM, neural network, or random forest) is used to detect the emotions. Some of the popular hand-crafted features used for facial expression recognition include the histogram of oriented gradients (HOG) [23,24], local binary patterns (LBP) [25], Gabor wavelets [26], and Haar features [27]. A classifier then assigns the best emotion to the image. These approaches seemed to work fine on simpler datasets, but with the advent of more challenging datasets (which have more intra-class variation), they started to show their limitations. To obtain a better sense of some of the possible challenges with the images, we refer the readers to the images in the first row of Figure 2, where the image shows only a partial face or the face is occluded with a hand or eyeglasses.

With the great success of deep learning and more specifically convolutional neural networks for image classification and other vision problems [28,29,30,31,32,33,34,35], several groups developed deep learning-based models for facial expression recognition (FER). To name some of the promising works, Khorrami in [17] showed that CNNs can achieve a high accuracy in emotion recognition and used a zero-bias CNN on the extended Cohn–Kanade dataset (CK+) and the Toronto Face Dataset (TFD) to achieve state-of-the-art results. Aneja et al. [2] developed a model of facial expressions for stylized animated characters based on deep learning by training a network to model the expression of human faces, one for that of animated faces, and one to map human images into animated ones. Mollahosseini [9] proposed a neural network for FER using two convolution layers, one max pooling layer, and four “inception” layers, i.e., sub-networks. Liu in [10] combined feature extraction and classification in a single looped network, citing the two parts’ needs for feedback from each other. They used their Boosted Deep Belief Network (BDBN) on CK+ and JAFFE, achieving state-of-the-art accuracy.

Barsoum et al. [36] used a deep CNN on noisy labels acquired via crowd-sourcing for ground truth images. They used 10 taggers to relabel each image in the dataset and used various cost functions for their DCNN, achieving decent accuracy. Han et al. [37] proposed an incremental boosting CNN (IB-CNN) in order to improve the recognition of spontaneous facial expressions by boosting discriminative neurons, which showed improvements over the best methods at the time. Meng in [38] proposed an identity-aware CNN (IA-CNN) that used identity- and expression-sensitive contrastive losses to reduce the variations in learning identity- and expression-related information. In [39], Fernandez et al. proposed an end-to-end network architecture for facial expression recognition with an attention model.

In [40], Want et al. proposed a simple yet efficient Self-Cure Network (SCN) that suppresses uncertainties efficiently and prevents deep networks from overfitting uncertain facial images (due to noisy labels). Specifically, SCN suppresses the uncertainty from two different aspects: (1) a self-attention mechanism over a mini-batch to weight each training sample with a ranking regularization and (2) a careful relabeling mechanism to modify the labels of these samples in the lowest-ranked group. In [41], Wang et al. developed a facial expression recognition algorithm that is robust to real-world pose and occlusion variations. They proposed a novel Region Attention Network (RAN) to adaptively capture the importance of facial regions for occlusion and pose variant FER. Some of the other recent works on facial expression recognition includes Multiple attention network for facial expression recognition [42], deep self-attention network for facial emotion recognition [43], and a recent survey on facial expression recognition [44].

All of the above works achieved significant improvements over traditional works on emotion recognition, but they are missing a simple method for recognizing important facial regions for emotion detection. In this work, we try to address this problem by proposing a framework based on an attentional convolutional network that is able to focus on salient facial regions.

## 3. The Proposed Framework

We propose an end-to-end deep learning framework based on an attentional convolutional network to classify the underlying emotion in facial images. Often times, improving a deep neural network relies on adding more layers/neurons, facilitating gradient flow in the network (e.g., by adding skip layers), or better regularizations (e.g., spectral normalization), especially for classification problems with a large number of classes. However, for facial expression recognition, due to the small number of classes, we show that using a convolutional network with less than 10 layers and attention (which is trained from scratch) is able to achieve promising results, presenting better results than state-of-the-art models for several databases.

Given a facial image, it is clear that not all parts of the face are important for detecting a specific emotion, and in many cases, we only need to pay attention to specific regions to get a sense of the underlying emotion. Based on this observation, we added an attention mechanism, through spatial transformer network into our framework to focus on important facial regions.

**Figure 3:**
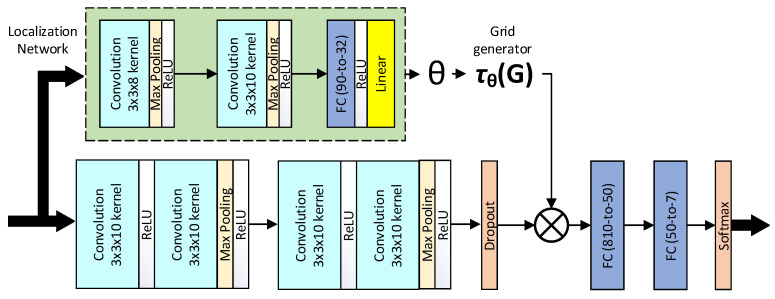
The proposed model architecture.

Figure 3 illustrates the proposed model architecture. The feature extraction part consists of four convolutional layers, with every two followed by a max-pooling layer and a rectified linear unit (ReLU) activation function. They were then followed by a dropout layer and two fully connected layers. The spatial transformer (the localization network) consisted of two convolution layers (each followed by max-pooling and ReLU) and two fully connected layers. After regressing the transformation parameters, the input was transformed to the sampling grid T(θ), producing the warped data. The spatial transformer module essentially tries to focus on the most relevant parts of the image by estimating a sample over the region of interest. One can use different transformations to warp the input to the output; here, we used an affine transformation, which is commonly used for many applications. For further details about the spatial transformer network, please refer to [45].

This model was then trained by optimizing a loss function using the stochastic gradient descent approach (more specifically, the Adam optimizer). The loss function in this work is simply the summation of two terms, the classification loss (cross-entropy), and the regularization term (which is the $\ell_{2}$ norm of the weights in the last two fully-connected layers).

(1) $$L_{overall}=L_{classifier}+\lambda {∥w_{\left(fc\right)}∥}_{2}^{2}$$

The regularization weight, λ, is tuned based on the model performance on the validation set to pick the corresponding value that yields the highest performance on the validation set. Adding both dropout and $\ell_{2}$ regularization enables us to train our models from scratch even on very small datasets, such as JAFFE and CK+. It is worth mentioning that we trained a separate model for each of the databases used in this work. We also tried using a network with similar architecture but more than 50 layers, but the accuracy did not improve significantly. Therefore, we decided to use the network with fewer layers, which has a much faster inference speed and is more suitable for real-time applications.

## 4. Experimental Results

In this section, we provide the detailed experimental analysis of our model on several facial expression recognition databases. We first provide a brief overview of the databases used in this work, then provide the performance of our models on four databases, and compare the results with some of the promising recent works. We then provide the salient regions detected by our trained model using a visualization technique.

### 4.1. Databases

In this work, we provide the experimental analysis of the proposed model on several popular facial expression recognition datasets, including FER2013 [14], the extended Cohn–Kanade [46], Japanese Female Facial Expression (JAFFE) [15], and Facial Expression Research Group Database (FERG) [2]. Before diving into the results, we give a brief overview of these databases.

**Figure 4:**
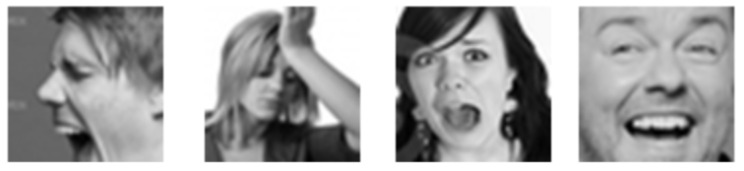
Four sample images from the FER database.

**FER2013**: The Facial Expression Recognition 2013 (FER2013) database was first introduced in the ICML 2013 Challenges in Representation Learning [14]. This dataset contains 35,887 images of 48 × 48 resolution, most of which are taken in wild settings. Originally, the training set contained 28,709 images, and the validation and test sets include 3589 images each. This database was created using the Google image search API, and faces were automatically registered. The faces are labeled as any of the six cardinal expressions as well as neutral. Compared to the other datasets, FER has more variation in the images, including facial occlusion (mostly with a hand), partial faces, low-contrast images, and eyeglasses. Four sample images from the FER dataset are shown in Figure 4.

**Figure 5:**
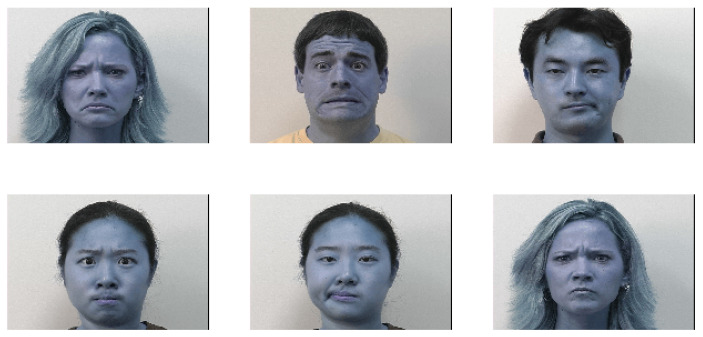
Six sample images from the CK+ database.

**CK+**: The extended Cohn–Kanade (known as CK+) facial expression database [46] is a public dataset for action unit and emotion recognition. It includes both posed and non-posed (spontaneous) expressions. The CK+ comprises a total of 593 sequences across 123 subjects. In most previous work, the last frame of these sequences is taken and used for image-based facial expression recognition. Six sample images from this dataset are shown in Figure 5.

**Figure 6:**
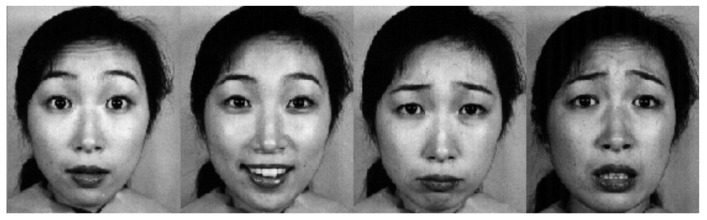
Four sample images from the JAFFE database.

**JAFFE**: This dataset contains 213 images of the 7 facial expressions posed by 10 Japanese female models. Each image has been rated on the six emotional adjectives by 60 Japanese subjects [15]. Four sample images from this dataset are shown in Figure 6.

**Figure 7:**
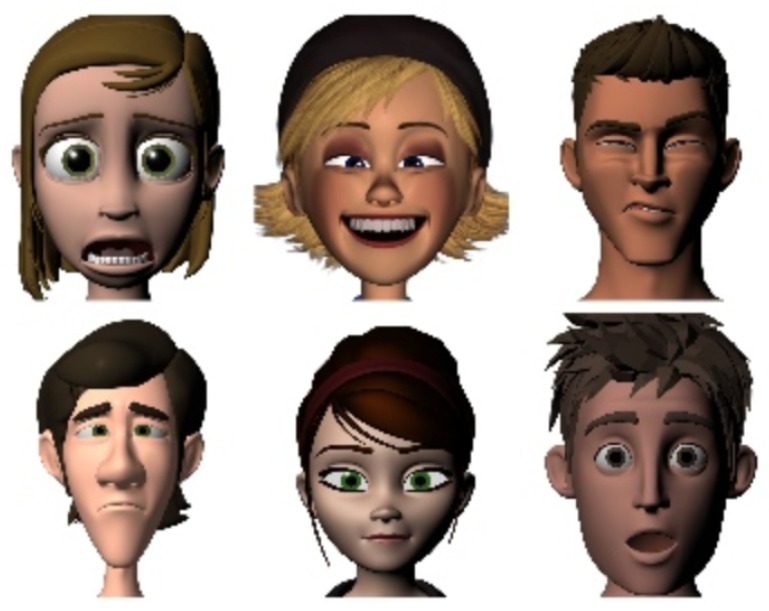
Six sample images from the FERG database.

**FERG**: FERG is a database of stylized characters with annotated facial expressions. The database contains 55,767 annotated face images of six stylized characters. The characters were modeled using MAYA. The images for each character are grouped into seven types of expressions [2]. Six sample images from this database are shown in Figure 7. We mainly wanted to try our algorithm on this database to see how it performs on cartoonish characters.

### 4.2. Experimental Analysis and Comparison

We now present the performance of the proposed model on the above datasets. In each case, we trained the model on a subset of that dataset, validated it on a validation set, and reported the accuracy over the test set.

Before getting into the details of the model’s performance on different datasets, we briefly discuss our training procedure. We trained one model per dataset in our experiments, but we tried to keep the architecture and hyperparameters similar among these different models. Each model was trained for 300 epochs from scratch, on an AWS EC2 instance with a Nvidia Tesla K80 GPU. We initialized the network weights with random Gaussian variables with zero mean and 0.05 standard deviation. For optimization, we used an Adam optimizer with a learning rate of 0.005 (different optimizers were used, including stochastic gradient descents, and Adam seemed perform slightly better). We also added L2 regularization with a weight decay value of 0.001. It took around 2–4 h to train our models on the FER and FERG datasets. For JAFFE and CK+, since there are much fewer images, it took less than 10 min to train a model. For datasets with large class imbalance, we used oversampling on the classes with fewer samples to force the different classes to be of the same order. Data augmentation was used for the images in the training sets to train the model on a larger number of images and to train model for invariances on small transformations. We used flipping, small rotation, and small distortion to augment the data.

As discussed before, the FER-2013 dataset is more challenging than the other facial expression recognition datasets we used. Besides the intra-class variation of FER, another main challenge in this dataset is the imbalanced nature of different emotional classes. Some of the classes such as happiness and neutral have many more examples than others. We used all 28,709 images in the training set to train the model, validated on 3500 validation images, and reported the model accuracy on the 3589 images in the test set. We were able to achieve an accuracy rate of around 70.02% on the test set.

The comparison of the result of our model with some of the previous works on FER 2013 is provided in Table 1.

For the FERG dataset, we used around 34,000 images for training, 14,000 for validation, and 7000 for testing. For each facial expression, we randomly select 1000 images for testing. We were able to achieve an accuracy rate of around 99.3%. The comparison between the proposed algorithm and some of the previous works on FERG dataset are provided in Table 2.

For the JAFFE dataset, we used 120 images for training, 23 images for validation, and 70 images for test (10 images per emotion in the test set). The overall accuracy on this dataset is around 92.8%. The comparison with previous works on the JAFFE dataset is shown in Table 3.

For CK+, 70% of the images were used as training, 10% was used for validation, and 20% was used for testing (which corresponds to 420, 60, and 113 images for the training, validation, and test sets, respectively). The comparison of our model with previous works on the extended CK dataset is shown in Table 4.

### 4.3. Confusion Matrix

**Figure 8:**
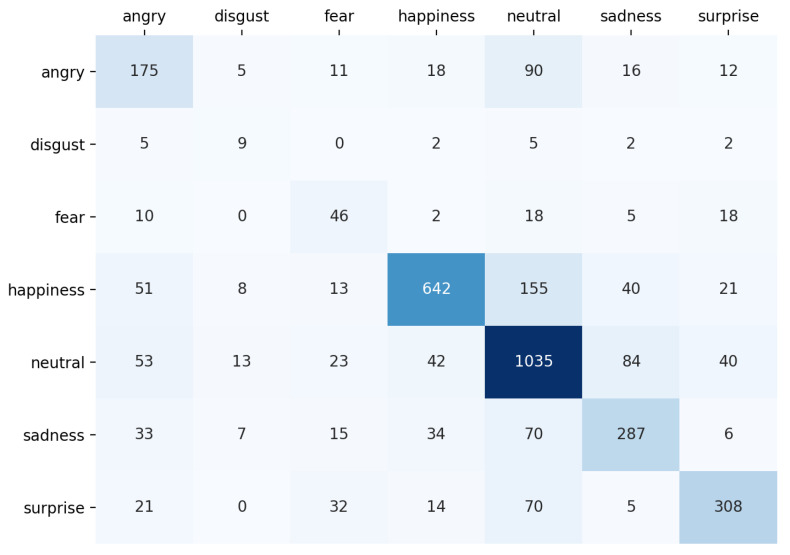
The confusion matrix of the proposed model on the test set of the FER dataset. The number of images for each emotion class in the test set is as follows: angry: 328, disgust: 25, fear: 99, happiness: 930, neutral: 1290, sadness: 450, and surprise: 450.

The confusion matrix of the proposed model on the test set of FER dataset is shown in Figure 8. As we can see, the model makes more mistakes for classes with less samples such as disgust and fear.

**Figure 9:**
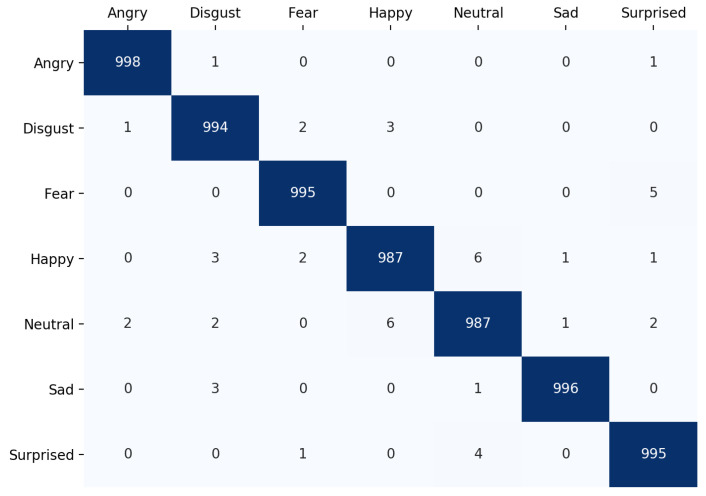
The confusion matrix on the FERG dataset. Each emotion class has 1000 images in the test set.

The confusion matrix of the proposed model on the test set of the FERG dataset is shown in Figure 9.

**Figure 10:**
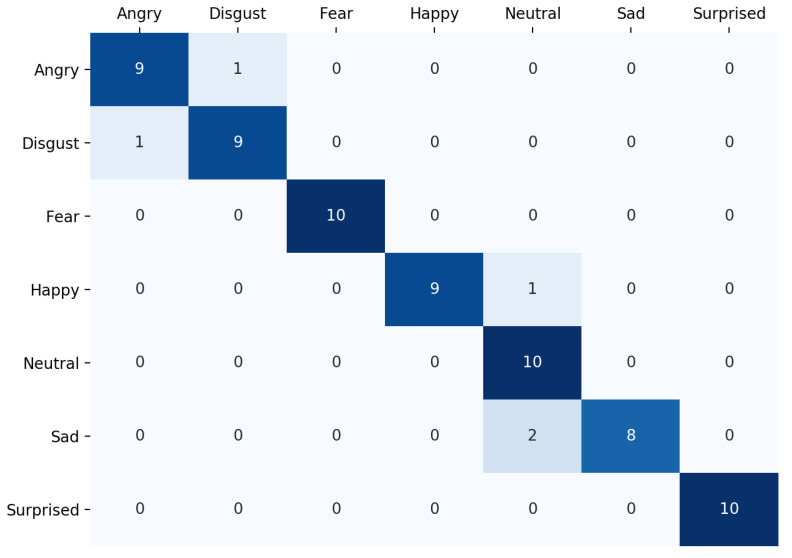
The confusion matrix on the JAFFE dataset. There are a total of seven emotions, and each class has 10 images in the test set.

The confusion matrix of the proposed model on the JAFFE dataset is shown in Figure 10.

### 4.4. Model Visualization

Here, we provide a simple approach for visualizing important regions while classifying different facial expressions, inspired by the work in [20]. We start from the top-left corner of an image, and each time, we zero out a square region of size N×N inside the image and make a prediction using the trained model on the occluded image. If occluding that region makes the model provide a wrong prediction in terms of facial expression label that region is considered a potential region of importance for classifying that specific expression. On the other hand, if removing that region does not impact the model’s prediction, we infer that region as being not very important in detecting the corresponding facial expression. Now, if we repeat this procedure for different sliding windows of N×N, each time shifting them with a stride of *s*, we can obtain a saliency map for the most important regions in detecting an emotion from different images.

**Figure 11:**
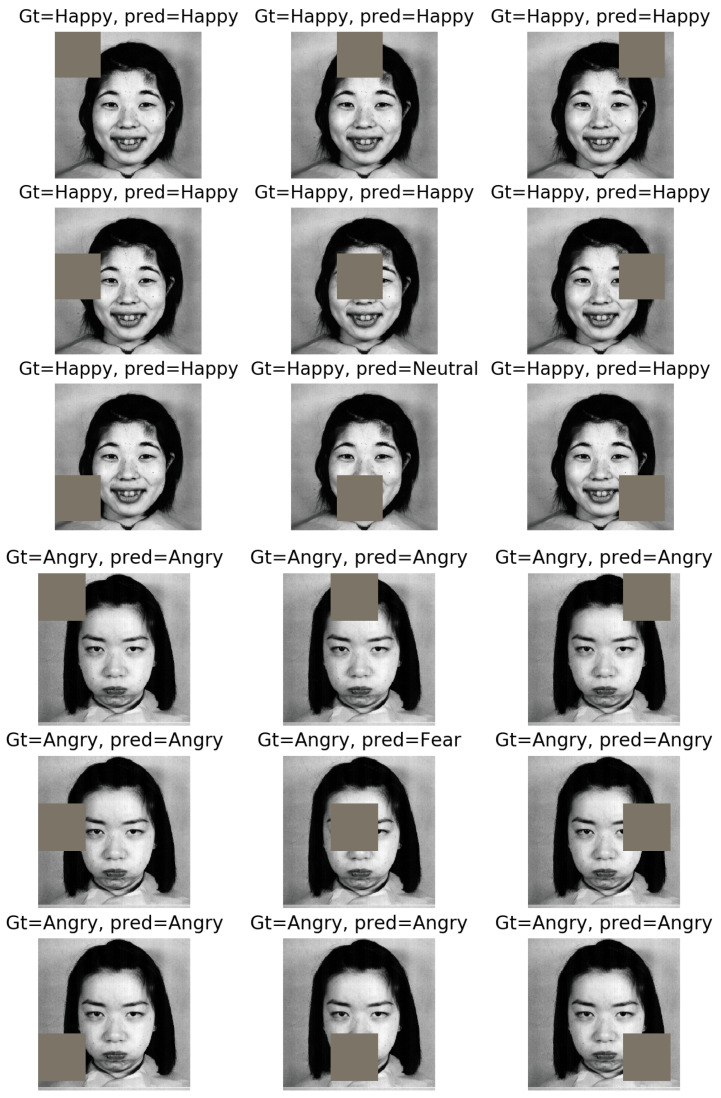
The impact of zeroing out different image parts on the model prediction for a happy face (**the top three rows**) and an angry face (**the bottom three rows**).

We show nine example cluttered images for a happy and an angry image from the JAFFE dataset and how zeroing out different regions would impact the model prediction in Figure 11. As we can see, for a happy face, zeroing out the areas around the mouth would cause the model to make a wrong prediction, whereas for an angry face, zeroing out the areas around eye and eyebrow makes the model make a mistake.

**Figure 12:**
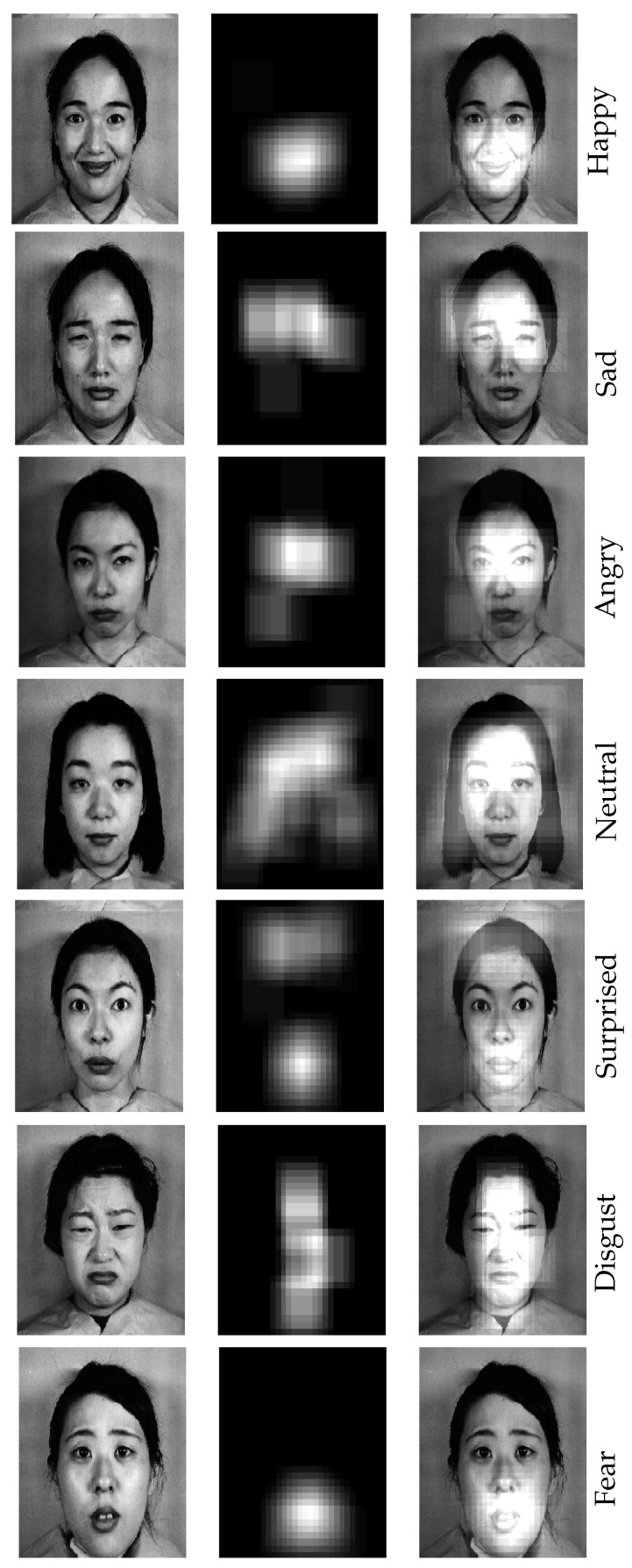
The important regions for detecting different facial expressions. As it can be seen, the saliency maps for happiness, fear, and surprise are sparser than that for the other emotions.

Figure 12 shows the important regions of seven sample images from the JAFFE dataset, each corresponding to a different emotion. There are some interesting observations from these results. For example, for the sample image with neutral emotion in the fourth row, the saliency region essentially covers the entire face, which means that all of these regions are important to infer that a given image has a neutral facial expression.

This makes sense, since changes in any part of the face (such as the eyes, lips, eyebrows, and forehead) could lead to a different facial expression, and the algorithm needs to analyze all of those parts in order to correctly classify a neutral image. This is however not the case for most of the other emotions, such as happiness, and fear, where the areas around the mouth turns out to be more important than other regions.

**Figure 13:**
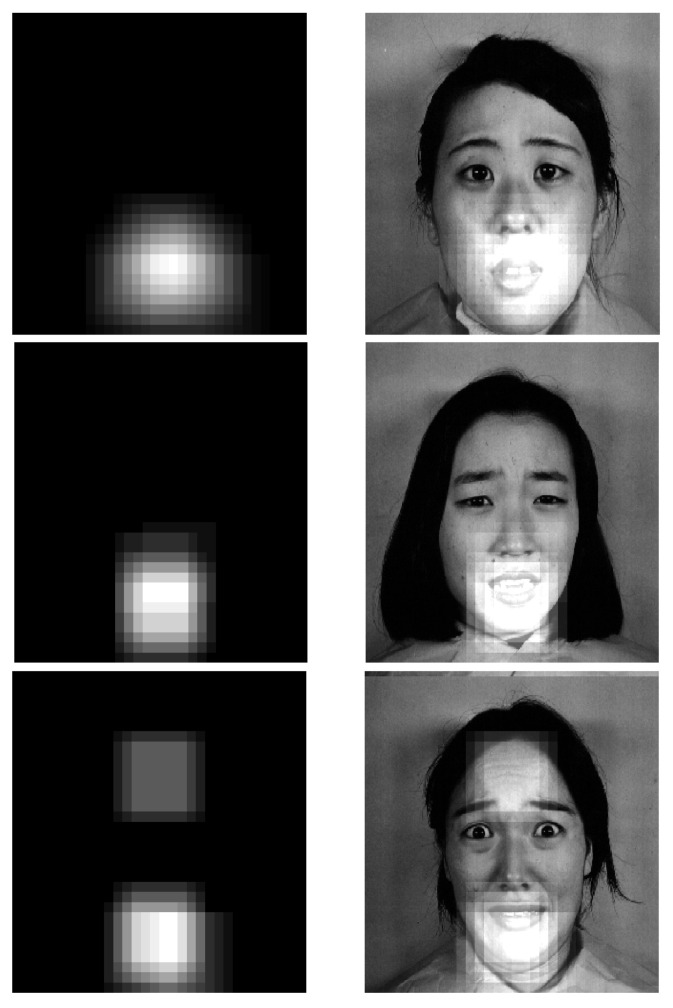
The important regions for three images of fear.

It is worth mentioning that different images with the same facial expression could have different saliency maps due to the different gestures and variations in the image. In Figure 13, we show the important regions for three images with a facial expression of “fear”. As seen in this figure, the important regions for these images are very similar when detecting the mouth, but the last one also considers some parts of forehead as important regions. This could be because of the strong presence of forehead lines, which is not visible in the two other images.

### 4.5. Model Convergence

**Figure 14:**
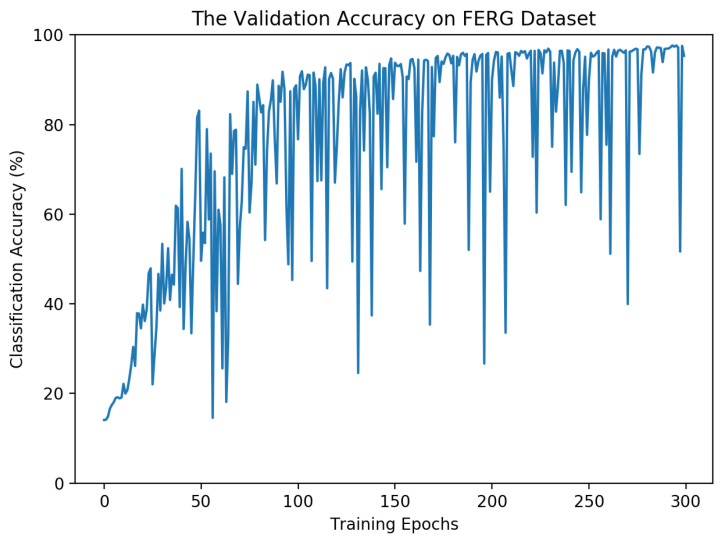
The validation accuracy over different epochs for the model trained on the FERG dataset.

In this part, we present the model classification accuracy on the validation set during the training. This helps us obtain a better understanding of the model convergence speed. The model performance on the validation set for the FERG dataset is presented in Figure 14. As seen, the general trend in validation accuracy increases over time but there are some oscillations at some epochs.

These oscillations could be avoided by choosing a smaller learning rate, but that could lead to slower convergence while training the model. It is worth mentioning that, in the end, the model with the highest validation accuracy is used to report the test error. Through experimental results, we noticed that the choice of learning rate is very important and that choosing a learning rate larger than 0.01 usually leads to model divergence. This could be because of the limited number of samples for some of the datasets.

## 5. Conclusions

This paper proposes a new framework for facial expression recognition using an attentional convolutional network. We believe attention to special regions is important for detecting facial expressions, which can enable neural networks with less than 10 layers to compete with (and even outperform) much deeper networks in emotion recognition. We also provide an extensive experimental analysis of our work on four popular facial expression recognition databases and show some promising results. Additionally, we deployed a visualization method to highlight the salient regions of face images that are the most crucial parts thereof for detecting different facial expressions.

## Figures and Tables

**Table 1:** Classification accuracies on the FER 2013 dataset.

Method	Accuracy Rate
Unsupervised Domain Adaptation [47]	65.3%
Bag of Words [48]	67.4%
VGG+SVM [49]	66.31%
GoogleNet [50]	65.2%
FER on SoC [51]	66%
Mollahosseini et al. [9]	66.4%
The proposed algorithm	70.02%
Aff-Wild2 (VGG backbone) [52]	75%

**Table 2:** Classification accuracy on the FERG dataset.

Method	Accuracy Rate
DeepExpr [2]	89.02%
Ensemble Multi-feature [53]	97%
Adversarial NN [54]	98.2%
The proposed algorithm	99.3%

**Table 3:** Classification accuracy on the JAFFE dataset.

Method	Accuracy Rate
LBP+ORB features [55]	88.5%
Fisherface [56]	89.2%
Deep Features + HOG [57]	90.58%
Salient Facial Patch [58]	91.8%
CNN+SVM [59]	95.31%
The proposed algorithm	92.8%

**Table 4:** Classification accuracy on CK+.

Method	Accuracy Rate
MSR [60]	91.4%
3DCNN-DAP [61]	92.4%
LBP+ORB features [55]	93.2%
Inception [9]	93.2%
Deep Features + HOG [57]	94.17%
IB-CNN [37]	95.1%
IACNN [38]	95.37%
DTAGN [62]	97.2%
ST-RNN [63]	97.2%
PPDN [64]	97.3%
Dynamic cascaded classifier [65]	97.8%
The proposed algorithm	98.0%

## Data Availability

Not applicable.
